# Integrated plasma metabolomic and cytokine analysis reveals a distinct immunometabolic signature in atopic dermatitis

**DOI:** 10.3389/fimmu.2024.1354128

**Published:** 2024-03-15

**Authors:** Emily Z. Ma, Junwen Deng, Varsha Parthasarathy, Kevin K. Lee, Thomas Pritchard, Shenghao Guo, Cissy Zhang, Madan M. Kwatra, Anne Le, Shawn G. Kwatra

**Affiliations:** ^1^ Department of Dermatology, University of Maryland School of Medicine, Baltimore, MD, United States; ^2^ Maryland Itch Center, University of Maryland School of Medicine, Baltimore, MD, United States; ^3^ Department of Dermatology, Johns Hopkins University School of Medicine, Baltimore, MD, United States; ^4^ Pathology, Johns Hopkins University School of Medicine, Baltimore, MD, United States; ^5^ Pharmacology and Cancer Biology, Duke University School of Medicine, Durham, NC, United States; ^6^ Anesthesiology, Duke University School of Medicine, Durham, NC, United States

**Keywords:** atopic dermatitis, itch, inflammation, metabolomics, cytokines, immunology

## Abstract

**Importance:**

Disease models for atopic dermatitis (AD) have primarily focused on understanding underlying environmental, immunologic, and genetic etiologies. However, the role of metabolic mechanisms in AD remains understudied.

**Objective:**

To investigate the circulating blood metabolomic and cytokine profile of AD as compared to healthy control patients.

**Design:**

This study collected plasma from 20 atopic dermatitis with moderate-to-severe itch (score of ≥5 on the itch Numeric Rating Scale and IGA score ≥3) and 24 healthy control patients. Mass-spectrometry based metabolite data were compared between AD and healthy controls. Unsupervised and supervised machine learning algorithms and univariate analysis analyzed metabolic concentrations. Metabolite enrichment and pathway analyses were performed on metabolites with significant fold change between AD and healthy control patients. To investigate the correlation between metabolites levels and cytokines, Spearman’s rank correlation coefficients were calculated between metabolites and cytokines.

**Setting:**

Patients were recruited from the Johns Hopkins Itch Center and dermatology outpatient clinics in the Johns Hopkins Outpatient Center.

**Participants:**

The study included 20 atopic dermatitis patients and 24 healthy control patients.

**Main outcomes and measures:**

Fold changes of metabolites in AD vs healthy control plasma.

**Results:**

In patients with AD, amino acids isoleucine, tyrosine, threonine, tryptophan, valine, methionine, and phenylalanine, the amino acid derivatives creatinine, indole-3-acrylic acid, acetyl-L-carnitine, L-carnitine, 2-hydroxycinnamic acid, N-acetylaspartic acid, and the fatty amide oleamide had greater than 2-fold decrease (all P-values<0.0001) compared to healthy controls. Enriched metabolites were involved in branched-chain amino acid (valine, leucine, and isoleucine) degradation, catecholamine biosynthesis, thyroid hormone synthesis, threonine metabolism, and branched and long-chain fatty acid metabolism. Dysregulated metabolites in AD were positively correlated cytokines TARC and MCP-4 and negatively correlated with IL-1a and CCL20.

**Conclusions and relevance:**

Our study characterized novel dysregulated circulating plasma metabolites and metabolic pathways that may be involved in the pathogenesis of AD. These metabolic pathways serve as potential future biomarkers and therapeutic targets in the treatment of AD.

## 1 Introduction

Atopic dermatitis (AD) is an intensely pruritic, inflammatory skin disease that has underlying environmental, immunologic, and genetic contributing factors (1, 2). The precise mechanism of AD is not fully understood but it is known that immune dysregulation plays a prominent role in disease biology and is the focus of multiple approved therapeutics and additional agents in therapeutic development. Cytokines from T helper 2 (Th2) cells have primarily been implicated in neuroinflammation, as well as focal inputs in patient subsets of Th1, Th17, and Th22 inflammation pathways (3, 4). To complement the evolving understanding of AD, we investigated circulating blood metabolic pathways that are dysregulated in AD. Metabolomic changes can modulate immune pathways, which was noted in a recent study on integrated cytokine and metabolite analysis revealing immunometabolic reprogramming in COVID-19 patients (5, 6). Plasma metabolomics studies have also found increased microbial-derived metabolites, such as dimethylamine and isopropanol in patients with AD (7). Our study aims to integrate plasma metabolite and cytokine profiling to analyze novel axes of AD pathogenesis and discover new biomarkers and therapeutic targets for AD.

## 2 Methods

### 2.1 Patient characteristics

Plasma from 20 AD patients and 24 healthy controls (HC) was collected for metabolomic analysis. Plasma from 19 AD and 33 HC patients were collected for cytokine analysis. There were 18 overlapping AD patients in the metabolomic and cytokine analysis cohorts. Different cohorts were used given the limitations in the number of plasma samples available for each experiment. The subjects’ demographic characteristics are summarized in **Supplementary Table S1**. For metabolomic analysis, a subset (12 AD, 12 HC) from initial samples was selected to compare sex- and race-matched groups. The demographic characteristics of the matched groups are also summarized in **Supplementary Table S1**.

Patients over the age of 18 were recruited from the dermatology outpatient clinics at Johns Hopkins Hospital from August 2018 to February 2020. Patients with AD were diagnosed by a board-certified dermatologist in accordance with the Hanifin and Rajka criteria (8). Adult AD patients with self-reported moderate-to-severe pruritus with a score of ≥5 on the itch Numeric Rating Scale (NRS) and IGA score ≥3 were included (9). HC patients were clinically determined to have no known cutaneous disease. Exclusion criteria included patients with systemic inflammatory conditions such as infectious, autoimmune, rheumatologic, severe atherosclerotic, and other inflammatory dermatologic diseases, and those receiving systemic immunosuppressive or immunomodulatory therapies.

### 2.2 Ethics

This study was reviewed and approved by the Institutional Review Board (IRB00231694). All participants signed written informed consent forms.

### 2.3 Metabolomic sample preparation and instrumentation

Sample preparation was performed at the Metabolomics Facility at the Johns Hopkins Medical Institution. Metabolites were extracted from plasma samples by adding high-performance liquid chromatography (HPLC)-grade methanol (Fisher Scientific) to a final concentration of 80% (vol/vol) methanol. Samples were centrifuged and supernatant was transferred for methanol evaporation using a speed vacuum. The entire sample was subsequently lyophilized. 50% (vol/vol) acetonitrile diluted in mass-spectrometry-(MS) grade water was then used to re-suspend the lyophilized metabolites. Metabolomics data acquisition was performed using a Thermo Scientific Q Exactive Plus Orbitrap Mass Spectrometer with a Vanquish UPLC system. The Vanquish UPLC auto-sampler systems were used to uptake 2ul of each sample, which was kept at 4°C. Reverse-phase chromatography was performed with 0.1% formic acid in mass spectrometry-grade water as the mobile aqueous phase and 0.1% formic acid in 98% acetonitrile as the mobile organic phase. A Discovery HS F5 HPLC Column (Sigma) together with a suitable guard column (Sigma) were used and maintained at 35°C. Metabolites were measured in both positive and negative polarities. If a compound is found in both polarities, then the one with better identification and quantification is presented. Full MS scans were performed for the quantification of metabolites and full MS/ddMS2 scans were acquired to identify metabolites via fragmentation pattern matching. Mass calibration was carried out before data acquisition. Quality control (QC) samples were analyzed to ensure system performance.

For all the metabolites, the identification is based on *m/z* accuracy and MS/MS fragmentation matching if available. All metabolites have MSI annotation level 2. For mass error, all metabolites have mass error of less than 5 ppm. Acquired data were analyzed using Thermo Scientific Compound Discoverer and Thermo Scientific TraceFinder software. A detailed table with the physio-chemical characteristics of the metabolites are summarized in **Supplementary Table S2**.

### 2.4 Cytokine sample preparation and instrumentation

The Bioplex 200 platform (Biorad, Hercules, CA) was used to determine the concentration of multiple target proteins in the extracted specimens. Luminex bead-based immunoassays (Millipore, Billerica, NY) were performed following Immune Monitoring Core SOPs and concentrations were determined using 5 parameter log curve fits (using Bioplex Manager 6.0) with vendor-provided standards and quality controls.

### 2.5 Statistical analysis

The raw intensities were normalized based on the protein concentration of each sample to obtain the normalized intensities (10, 11). MetaboAnalyst 5.0 (Quebec, Canada) was used for peak intensity noise filtering, data transformation, and scaling (12). Statistical analysis of the normalized intensities was performed using the Student’s t-test for continuous variables, and fold change intensity was obtained by dividing the normalized intensity of each sample by the average normalized intensity of the HC group. False discovery rate (FDR) corrections were applied to metabolite analyses. Multivariate analyses, including unsupervised and supervised machine learning techniques with principal component analysis (PCA), partial least squares discriminant analysis (PLS-DA), orthogonal partial least squares-discriminant analysis (OPLS-DA), and K-means clustering were performed to distinguish clusters of patients. MetaboAnalyst 5.0 was applied to create receiver operating characteristic (ROC) curves to identify potential biomarkers. To identify metabolite enrichment sets, the Small Molecule Pathway Database (SMPDB) of human metabolic pathways was used, and the analysis of metabolic pathways used the Kyoto Encyclopedia of Genes and Genomes (KEGG) Homo sapiens pathway library.

All statistical analyses were performed using R or MetaboAnalyst 5.0. Continuous and categorical variables were compared with a 2-tailed Student’s t-test and Pearson’s χ^2^, respectively. All data means are reported as mean ± standard deviation (SD) or standard error of the mean (SEM).

For the cytokine data, values below the detection limit (DL) were replaced by half the lower detection limit. Markers with >33% samples below the DL were dichotomized into binomial variables with values below DL as negative and values above DL as positive. To investigate the correlation between metabolites levels and cytokines, Spearman’s rank correlation coefficients were calculated between metabolites and cytokines. The heatmaps of data correlation between cytokines and metabolites were performed using Graphpad Prism 9.

Our study design is summarized in **Supplementary Figure S1**. Metabolomic and cytokine raw data are uploaded to Harvard Dataverse (13).

## 3 Results

### 3.1 Untargeted analysis of metabolite profiles in AD and HC plasma

**Figure 1:**
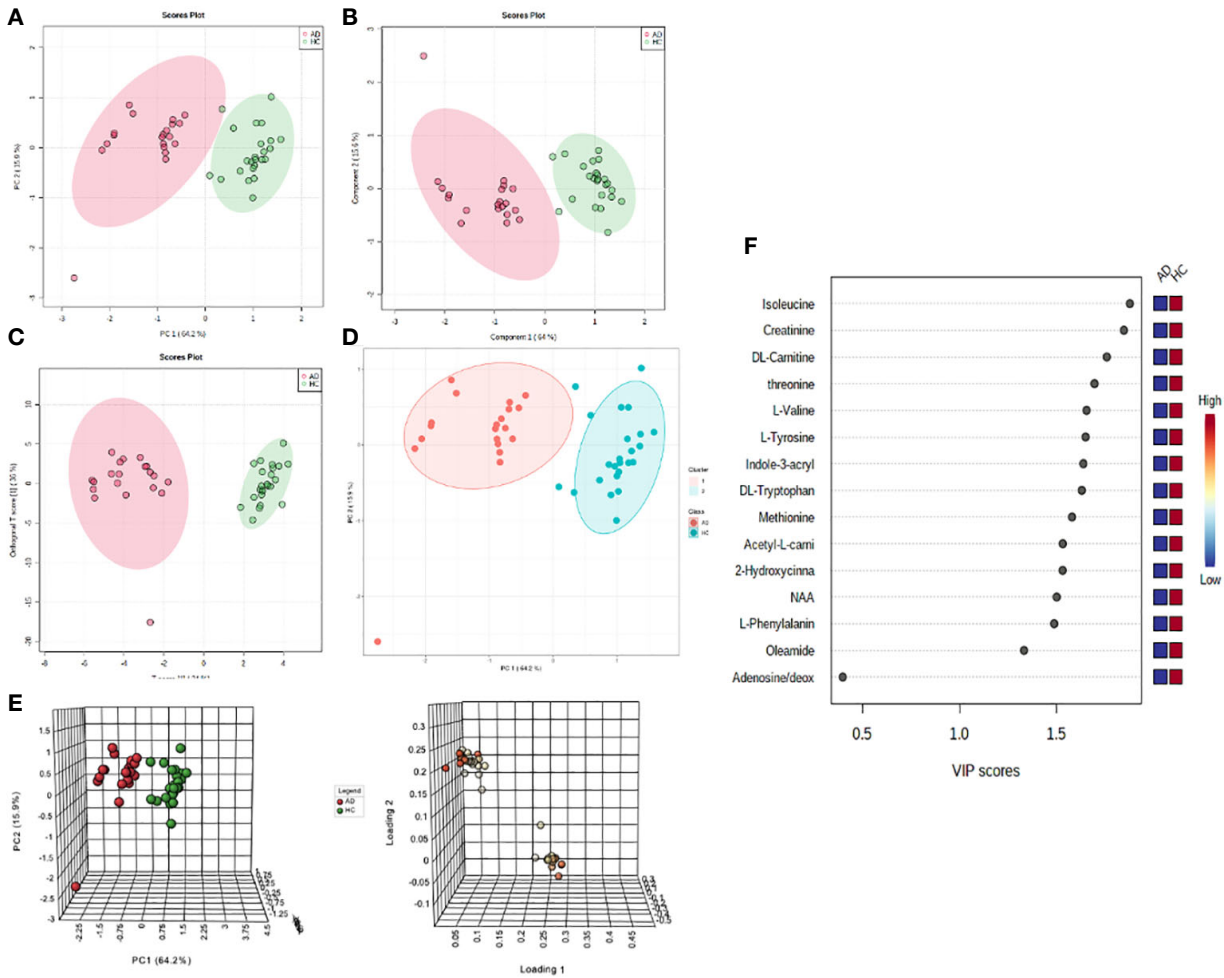
Non-targeted metabolomics profiling analysis of the plasma of atopic dermatitis (AD) patients and healthy controls (HC). For all scores plots, red=AD and green=HC. **(A)** PCA score plot with the first two components explaining for 80.1% of the variance. **(B)** PLS-DA score plot with the first two components explaining for 79.6% of the variance. **(C)** OPLS-DA score plot with the first two components explaining for 70% of the variance. **(D)** K-means clustering of groups. **(E)** Synchronized 3D scores and loading plots for visualization of the most influential compounds of the first three components on PCA. **(F)** Variable importance in projection (VIP) score ranking the most important metabolites.

Unsupervised (PCA, K-means clustering) and supervised (PLS-DA, OPLS-DA) machine learning algorithms were applied to the normalized and noise-filtered peak intensity data from matched AD and HC plasma samples (**Figure 1**). PCA analysis (**Figure 1A**), PLS-DA (**Figure 1B**), OPLS-DA (**Figure 1C**), and K-means clustering (**Figure 1D**) all resulted in clearly distinguished groups containing AD and HC individuals. Permutation tests of PLS-DA and OPLS-DA (**Supplementary Figure S2**) with 2000 iterations each revealed R^2^ and Q^2^ parameters of 0.88 and 0.82 (P<0.0005) and 0.94 and 0.85 (P<0.0005), respectively, validating the models’ strong predictive abilities and no overfitting. Synchronized 3D plots (**Figure 1E**) identify the clusters on the 3D scores plot (left) and the corresponding loadings plot (right) depicting the most influential metabolites of the clusters lying along the upper-right and lower-left most quadrants. The most important metabolites are depicted based on their variable importance in the projection score (**Figure 1F**).

**Figure 2:**
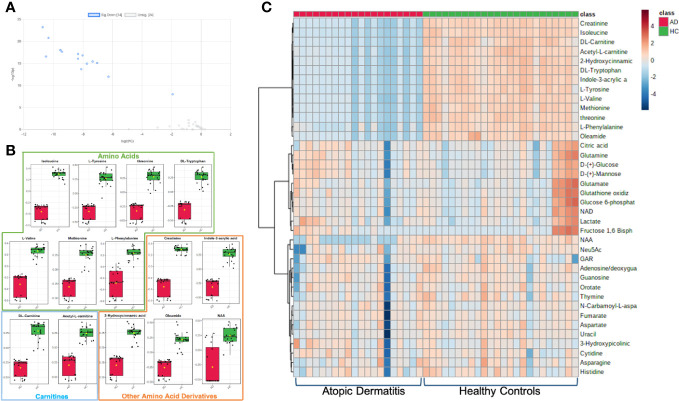
Metabolites with significant alterations in unmatched AD vs HC patients. **(A)** Volcano plot of significant fold change distribution of selected metabolites in AD versus HC (blue=significantly downregulated). **(B)** Boxplot summaries of the peak intensities of the significant metabolites (red=AD, green=HC). **(C)** Heatmap of the differential metabolites quantified in plasma in AD vs HC individuals using Euclidian distance measure and Ward clustering algorithm.

To identify the metabolites with significant alterations in AD versus HC groups, a volcano plot was created to compare metabolite fold changes with their P-values obtained from unpaired t-tests (**Figure 2A**). Significant molecules in AD patients that had greater than 2-fold change include the amino acids isoleucine, tyrosine, threonine, tryptophan, valine, methionine, and phenylalanine, the amino acid derivatives creatinine, indole-3-acrylic acid, acetyl-L-carnitine, L-carnitine, 2-hydroxycinnamic acid, N-acetylaspartic acid (NAA), and the fatty amide, oleamide (all P-values<.0001) (**Table 1**). The boxplot summary of peak intensities of significant metabolites (**Figure 2B**) and heatmap of differential metabolites quantified in AD and HC plasma corroborated these findings (**Figure 2C**). Among all our tested metabolites (**Supplementary Table S3**), none were found to be upregulated in AD patients. These significantly decreased metabolites were not found to be significantly correlated with itch NRS (**Supplementary Figure S3**).

**Table 1:** Metabolites in atopic dermatitis patients with greater than two-fold change.

	Metabolite description	Fold change	P-value^1^	FDR^2^
Isoleucine	amino acid	0.00058913	5.75E-24	2.19E-22
Creatinine	AA derivative	0.00077188	1.53E-21	2.90E-20
L-Tyrosine	amino acid	0.0013548	9.59E-19	1.22E-17
Threonine	amino acid	0.0014552	2.15E-18	2.05E-17
Indole-3-acrylic acid	AA (tryptophan) derivative	0.0031016	6.99E-18	5.31E-17
DL-Tryptophan	amino acid	0.0038264	1.46E-17	9.23E-17
DL-Carnitine	AA (lysine) derivative	0.00069027	2.60E-17	1.41E-16
L-Valine	amino acid	0.0030976	8.43E-17	4.01E-16
Acetyl-L-carnitine	carnitine derivative	0.0061407	3.47E-16	1.47E-15
2-Hydroxycinnamic acid	AA (phenylalanine/tyrosine) derivative	0.0075836	7.80E-16	2.97E-15
Methionine	amino acid	0.0046888	1.09E-15	3.76E-15
Oleamide	fatty amide	0.0036038	1.90E-14	6.00E-14
L-Phenylalanine	amino acid	0.012919	9.97E-13	2.91E-12
NAA	AA (aspartate) derivative	0.26288	9.51E-09	2.58E-08

Receiver operating characteristic (ROC) curve analysis was applied to assess the biomarker performance of the metabolites. The metabolites with the area under the curve (AUC) values between 0.9-1.0 represent excellent and robust biomarker performances, achieving the highest sensitivities and specificities, and those with AUC values between 0.8-0.9 represent good biomarker performances (14). The significant metabolites identified on the volcano plot and heatmap all correspond as being good- and excellent-performing biomarkers (**Supplementary Table S4**, **Supplementary Figure S4**).

To account for intrinsic demographic differences in metabolite levels, a similar gender-, age-, and race-matched pair analysis of a subset from the original samples was conducted (**Supplementary Figure S5**, **Supplementary Tables S5**, **S6**). In the resulting analysis, all the other identified metabolites remained significantly decreased to similar degrees as found in the unmatched analyses except creatinine. This may be explained by the older mean age of the unmatched HC group (47.58 ± 16.64 years) compared to the mean age of the unmatched AD group (40.3 ± 17.41 years), as older patients tend to have higher serum creatinine (15). This finding reduces the potential of creatinine as a biomarker for AD.

### 3.2 Metabolite enrichment and pathway analysis

**Figure 3:**
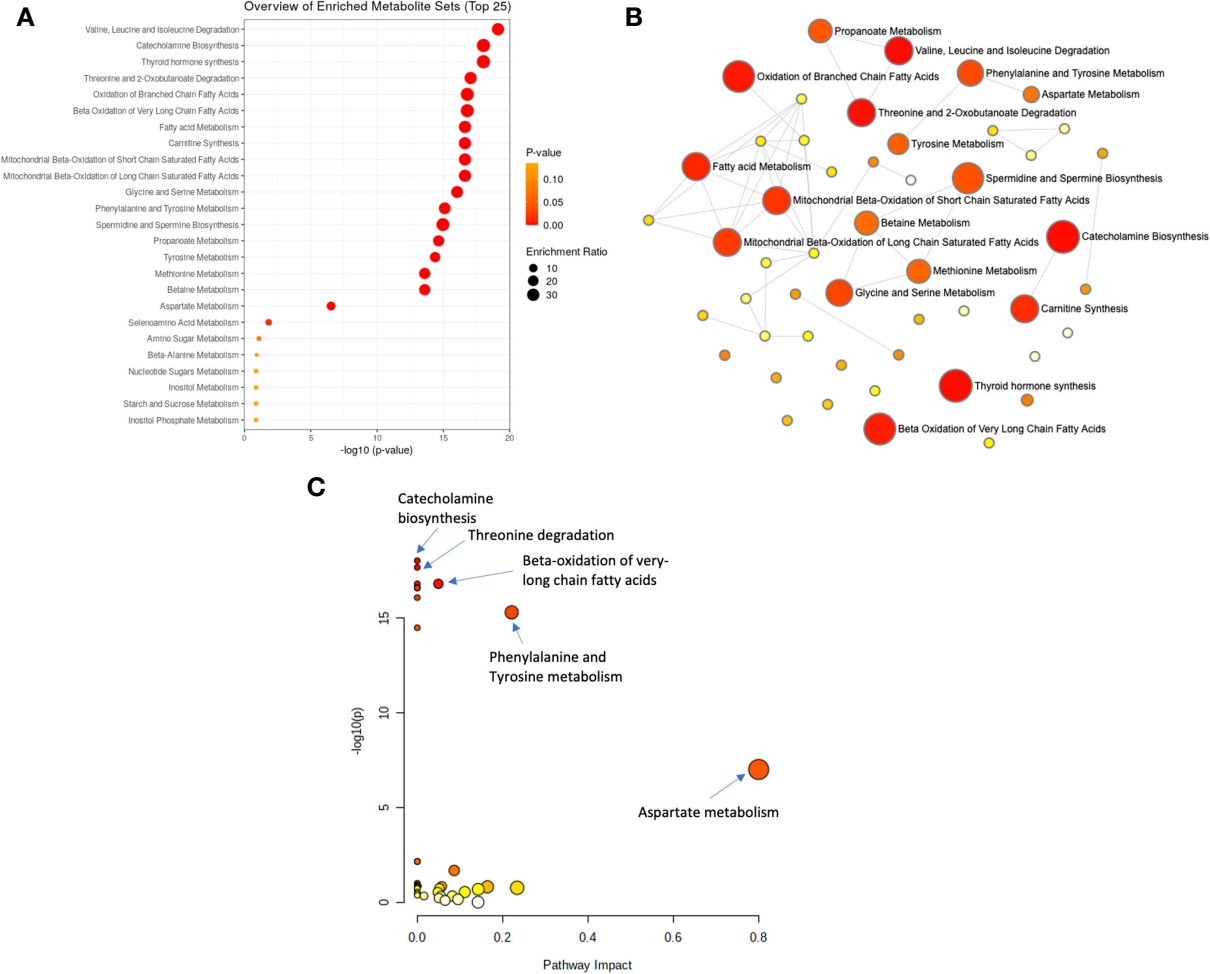
Analysis of significantly enriched metabolite sets. **(A)** Metabolic enrichment analysis based on the Small Molecule Pathway Database (SMPDB) of human metabolic pathways, with larger, darker-colored circles representing more significant metabolite sets with higher enrichment ratios. **(B)** Enrichment network view with each node representing a metabolite set, with its color corresponding to its P-value and its size representing its fold enrichment (hits/expected) to the database query. **(C)** Metabolic pathway analysis based on SMPDB, with larger, darker-colored circles representing more significant pathways with higher pathway impacts.

To explore the functional enrichment of the significant differential metabolites, we searched the Small Molecule Pathway Database (SMPDB) of human metabolic pathways on MetaboAnalyst 5.0. As shown in **Figure 3**, enriched metabolites were involved in branched-chain amino acid (valine, leucine, and isoleucine) degradation, catecholamine biosynthesis, thyroid hormone synthesis, threonine metabolism, branched and long-chain fatty acid metabolism, and more. Furthermore, metabolic pathway analysis showed that the differentially expressed metabolites in AD patients were enriched in pathways that similarly included catecholamine biosynthesis, fatty acid metabolism, and the metabolism of amino acids such as phenylalanine tyrosine, aspartate, threonine, glycine, and serine (**Supplementary Table S7**).

### 3.3 Metabolite and cytokine correlation analysis

**Figure 4:**
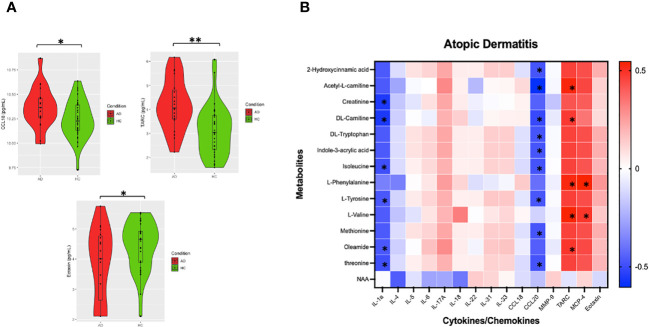
Analysis of AD metabolite levels and cytokines. **(A)** Violin plots of imputed and Ln transformed AD vs HC data. Single asterisk (*) indicates significance of p<.05, double asterisks (**) indicate significance of p<.001. **(B)** Correlation heatmap was made using Spearman’s rank values. Asterixis (*) represent Spearman’s rank-values between 0.4 and 1 (for positive correlation) or between (− 0.4 and − 1) with p-values < 0.05.

In our unmatched cohort cytokine analysis, t-tests showed TARC, CCL18, and eotaxin upregulation in AD compared to controls (**Figure 4A**). In samples from 18 AD patients with both cytokine and metabolite data, dysregulated metabolites were significantly positively associated with several cytokines, such as TARC and MCP-4, and negatively correlated multiple cytokines, including IL-1a and CCL20 (**Figure 4B**, **Supplementary Figure S6**). Correlation heatmaps summarized the major clusters of immunometabolic correlation in patients with AD (**Figure 4B**).

## 4 Discussion

Our study identified several dysregulated circulating blood metabolites in AD that are associated with systemic immune dysregulation. Pathway analysis revealed that the metabolic derangements in AD had significant effects on crucial cellular processes such as mitochondrial oxidation of branched chain and very long chain fatty acids, phenylalanine and tyrosine metabolism, carnitine biosynthesis, branched chain amino acid degradation, and catecholamine. Furthermore, decreased metabolites in AD were positively associated with TARC and MCP-4 and negatively correlated with IL-1a and CCL20. These findings suggest that therapies involved in immunometabolic reprogramming should be further explored as a novel therapeutic option in AD patients.

Previous studies have also observed differences in the blood metabolites in patients with and without AD (16). Targeted serum metabolomics have reported a decrease in acylcarnitine and phosphatidylcholines in patients with AD compared to healthy controls (17). Decreased sphingomyelin and acylcarnitine was also found in filaggrin (FLG) wild-type compared to FLG-mutant patients with AD (18). In separate untargeted plasma metabolic study, iso-butyrate, isoleucine, tyramine, histidine, threonine, and isopropanol were associated with FLG mutations, while isopropanol was associated with increased IgE levels (7) in AD.

Our study found that L-carnitine and its derivative acetyl-L-carnitine were significantly decreased in AD patients. Carnitines play essential roles in energy metabolism by transporting long-chain fatty acids from the cytosol to the mitochondrial matrix for β-oxidation (19). The lower levels of carnitine seen in AD patients could be due to increased in fatty acid oxidation in skin lesions from increased energy requirements in rapidly proliferating cells, which is a hypothesized mechanism explaining low carnitine levels in psoriasis patients (20, 21). Carnitine has been shown to have a protective effect in multiple systemic inflammatory disorders, including type 2 diabetes and cancer (22, 23). Single-nucleotide polymorphisms of the carnitine/organic cation transporter genes have also been associated with increased incidences of inflammatory diseases such as Crohn’s disease and asthma, although the mechanisms are not clear (24, 25). Given the strong association of AD with inflammatory disorders, L-carnitine may be a promising therapeutic target for the treatment of AD.

Alterations of amino acids are common in metabolic and inflammatory disorders. In this study, several essential amino acids (i.e., isoleucine, threonine, valine, tryptophan, methionine, and phenylalanine) were decreased in AD. Enhanced amino acid catabolism commonly occurs in response to inflammation (26). Tryptophan catabolism via the enzyme indoleamine 2,3-dioxygenase has been shown to be induced at inflammatory lesions and regulates T-cell-mediated immunity (26). Additionally, the decreased amino acids tryptophan, phenylalanine, and tyrosine are also involved in the biosynthesis of neurotransmitters. Tryptophan is converted to serotonin, whereas phenylalanine and tyrosine are converted to dopamine, melanin, and catecholamines. Catecholamines have been suggested to exert tonic inhibition on itch signaling in the spinal cord through action on α2-adrenergic receptors (27). In murine models, the administration of selective serotonin reuptake inhibitors (SSRIs) and selective norepinephrine serotonin reuptake inhibitors (SNRIs) attenuated the scratching behavior of mice with chronic itch, suggesting that these metabolites are involved in mitigating pruritus (28). In human clinical trials, SSRIs were found to have a substantial antipruritic effect in patients with chronic itch (29). The application of SSRIs and SNRIs in patients with AD could be an avenue for future therapeutic treatment.

The reduced levels of tryptophan in seen in AD patients may contribute to the decreased levels of its derivative, indole-3-acrylic acid (IAA). IAA is produced from tryptophan by commensal *Peptostreptococcus* bacteria in the gut microbiota (30), and its decreased levels in patients with AD may suggest an alteration in tryptophan metabolism or in the gut microbiome. In the skin-gut axis model, the gut microbiome is linked to the skin, as both organ systems are richly supplied with related immune and neuroendocrine functions. IAA has been proposed to promote intestinal epithelial barrier integrity and reduce inflammatory responses by immune cells (30). Thus, probiotics consisting of healthy gut microbial strains have been suggested as a potential preventative treatment for AD (31). Like tryptophan and other indole metabolites, IAA can bind to AHRs in the gastrointestinal tract (32). AHR has been shown to be involved in transcriptional regulation of both T regulatory (Treg) and Th17 cell differentiation (33), and depending on the particular receptor ligand, AHR can either accelerate or attenuate inflammation (34). The study of enzymes and metabolites involved in AHR pathways has uncovered various therapeutic targets in AD (35).

Other altered metabolites or enriched metabolic pathways in our AD patients include 2-hydroxycinnamic acid and oleamide. 2-hydroxycinnamic acid is a derivative of phenylalanine and tyrosine. Its homeostatic regulation in human plasma is not well-understood, but hydroxycinnamic acid derivatives serve important anti-inflammatory functions, particularly in obese animals, via modulating nuclear factor κB (NF-κB), TNF-α, and other inflammatory pathways (36). In murine models, oral administration of hydroxycinnamic acid has been found to attenuate the cutaneous and immunologic manifestations of AD, including pro-inflammatory cytokine production and serum immunoglobulin production (37). Oleamide is a signaling molecule in the central nervous system; however, not much is known about its effects or roles in immunity or cutaneous disease, although some animal studies have shown that oleamide has anti-inflammatory activity in glial cells and in counteracting lung inflammation (38).

Enriched metabolites in our study were involved in branched and long-chain fatty acid metabolism. Similarly, several Th2 cytokines associated with AD, such as IL-4, IL-5, IL-13, IL-25, and IL-33, have also been implicated in lipid metabolism (39–42). *In vitro* studies have shown that IL-4/IL-13 inhibit fatty acid elongases in a STAT6-dependent manner, which decreases long-chain fatty acids in AD (43). Additionally, the mTOR complex integrates signals from growth factors and cytokine receptors to regulate amino acid and lipid metabolism to promote Th2 cell differentiation, which consequently produce AD-associated cytokines (42). Animal studies have also shown that IL-4 regulates lipid metabolism by promoting glucose tolerance and inhibiting lipid deposits (40).

We also observed that cytokines TARC and CCL18 were upregulated in AD. CCL17/TARC and CCL18/PARC are Th2-related chemokines correlating with AD severity (44). Elevated serum CCL17 is seen in other inflammatory skin conditions including bullous pemphigoid and cutaneous T-cell lymphoma. In our AD patients, downregulated metabolites were significantly positively associated with TARC and MCP-4 and negatively correlated with IL-1a and CCL20, cytokines that have been previously implicated in the pathogenesis in AD. MCP-4, or monocyte chemotactic protein 4, is up-regulated at sites of allergic inflammation and is an eosinophil chemoattractant in AD (45). CCL20, a chemokine important for the recruitment of Th17 cells, is suppressed in the lesions of atopic dermatitis (46). IL-1a is a proinflammatory cytokine dysregulated in atopic dermatitis. Together, these findings suggest that there may be shared immune-metabolomic pathways involved in the pathogenesis of AD.

The strengths of the study include the usage of a matched pairs metabolomic data analysis to account for potential confounders such as age, sex, and race. Limitations of this study include the small sample size. Furthermore, the discussed metabolomic mechanisms are correlative, not causative, and thus may not represent exact mechanisms for the alterations of the identified metabolites. Given that alterations in the amino acids are common in inflammatory disorders, further studies comparing AD with other inflammatory skin diseases, systemic inflammatory diseases, and non-inflammatory pruritic diseases are needed. Despite these limitations, we were able to identify significantly altered metabolic pathways and biomarkers for AD, which builds a framework for further examination of AD metabolome and identification of therapeutic targets.

## 5 Conclusion

This study demonstrates the capabilities of metabolomics studies to identify significant metabolic pathways and biomarkers for AD. Our results suggest that carnitine, essential amino acids, and amino acid derivatives are downregulated in AD patients and are associated with inflammatory cytokine dysregulation, suggesting their role in the pathogenesis of AD and in regulating inflammation. Further studies are needed to study if these metabolomic alterations can predict AD risk or disease severity and to investigate immunometabolic reprogramming as a novel therapeutic approach in AD.

## Data availability statement

The original contributions presented in the study are publicly available. This data can be found here: https://doi.org/10.7910/DVN/VWLDUM.

## Ethics statement

This study was reviewed and approved by the Johns Hopkins School of Medicine Institutional Review Board (IRB) (IRB00231694). All participants signed written informed consent forms and provided blood samples. All experiments were conducted in accordance with the institution’s guidelines and regulations. The studies were conducted in accordance with the local legislation and institutional requirements. The participants provided their written informed consent to participate in this study.

## Author contributions

EM: Conceptualization, Formal analysis, Visualization, Writing – original draft, Writing – review & editing. JD: Conceptualization, Formal analysis, Validation, Writing – original draft. VP: Investigation, Writing – review & editing. KL: Investigation, Writing – review & editing. TP: Project administration, Writing – review & editing. SG: Investigation, Writing – review & editing. CZ: Investigation, Writing – review & editing. MK: Supervision, Writing – review & editing. AL: Investigation, Writing – review & editing. SK: Conceptualization, Funding acquisition, Resources, Supervision, Writing – review & editing.

## Supplementary material

Figure S1 A schematic of the study design. Plasma was collected from atopic dermatitis and healthy control patients and assayed for a panel of metabolites and cytokines. Metabolite and cytokine correlation analysis was performed. Unsupervised and supervised machine learning algorithm and univariate analysis were used on the metabolic concentrations to identify distinct clusters of AD. Metabolite enrichment and pathway analyses were then performed.

Figure S2 Validation plots for predictive ability of PLS-DA and OPLS-DA models. (A) PLS-DA validation plot for predictive ability, obtained from 2,000 permutation tests (at 2 components, R2=.88, Q2=.82, P<0.0005). (B) OPLS-DA validation plot for predictive ability obtained from 2,000 permutation tests (at 2 components, R2=0.94, Q2=.85, P<0.0005).

Figure S3 Scatterplots of Log(intensities) vs. itch numerical rating scores (INRS) of selected metabolites. Scatterplots of Log(intensities) vs. INRS scores of significantly decreased metabolites were not found to be significantly correlated with INRS scores.

Figure S4 Selected receiver operating characteristic (ROC) curves for biomarker analysis in unmatched patients. Selected ROC curves for metabolites with area under the curve (AUC) values equal to or approximately equal to 1.0, indicating their strong performance potentials as biomarkers.

Figure S5 Metabolites with significant alterations in matched AD vs HC patients. (A) Volcano plot of significant fold change distribution of selected metabolites in AD versus HC (blue=significantly downregulated). (B) Boxplot summaries of the peak intensities of the significant metabolites (red=AD, green=HC). (C) Heatmap of the differential metabolites quantified in plasma in AD vs HC individuals using Euclidian distance measure and Ward clustering algorithm.

Figure S6 Metabolite and cytokine correlation plots in AD patients. Dot plots summarizing the significant metabolite and cytokine correlations in patients with AD.
