# Supplementary figures and images for: Integrated plasma metabolomic and cytokine analysis reveals a distinct immunometabolic signature in atopic dermatitis
Source: Front Immunol. 2024 Mar 15;15:1354128. doi: 10.3389/fimmu.2024.1354128 (PMC10978712; doi:10.3389/fimmu.2024.1354128)

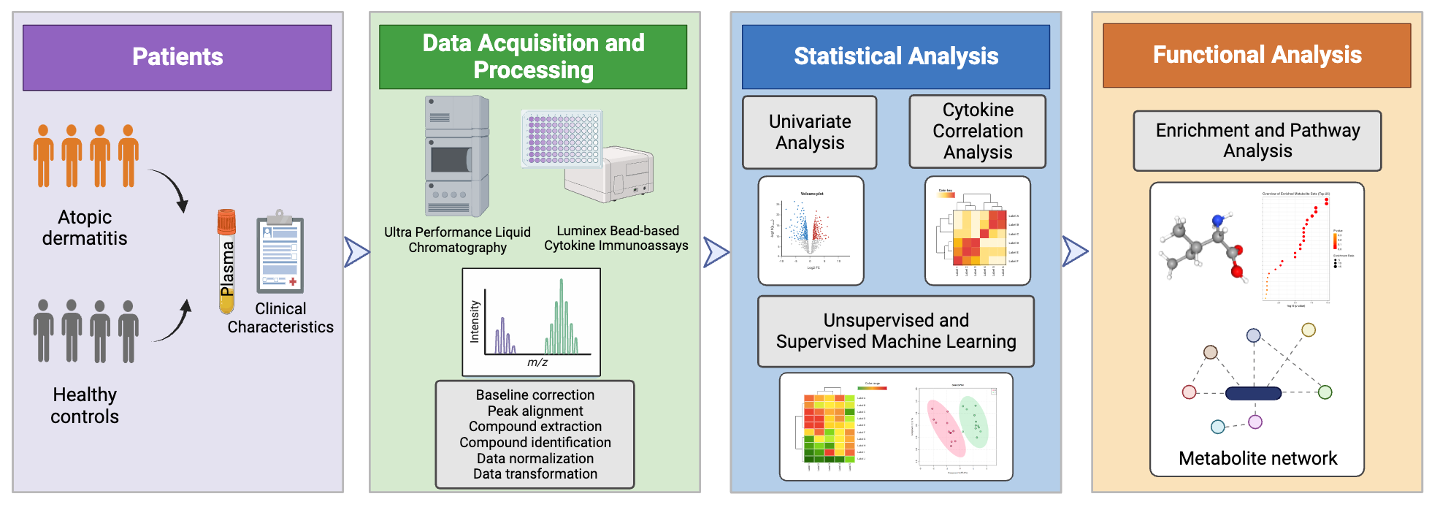

Supplement: Figure S1 — A schematic of the study design. Plasma was collected from atopic dermatitis and healthy control patients and assayed for a panel of metabolites and cytokines. Metabolite and cytokine correlation analysis was performed. Unsupervised and supervised machine learning algorithm and univariate analysis were used on the metabolic concentrations to identify distinct clusters of AD. Metabolite enrichment and pathway analyses were then performed. [file Image_1.jpeg]

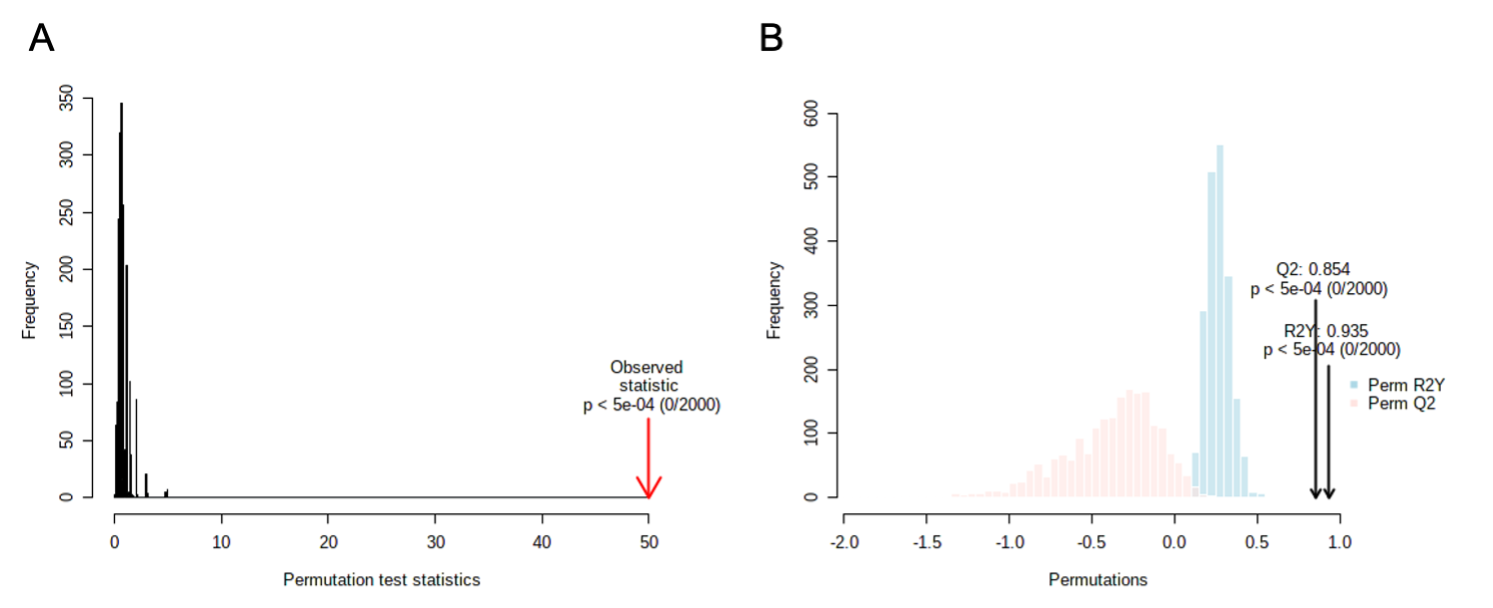

Supplement: Figure S2 — Validation plots for predictive ability of PLS-DA and OPLS-DA models. (A) PLS-DA validation plot for predictive ability, obtained from 2,000 permutation tests (at 2 components, R2=.88, Q2=.82, P<0.0005). (B) OPLS-DA validation plot for predictive ability obtained from 2,000 permutation tests (at 2 components, R2=0.94, Q2=.85, P<0.0005). [file Image_2.jpeg]

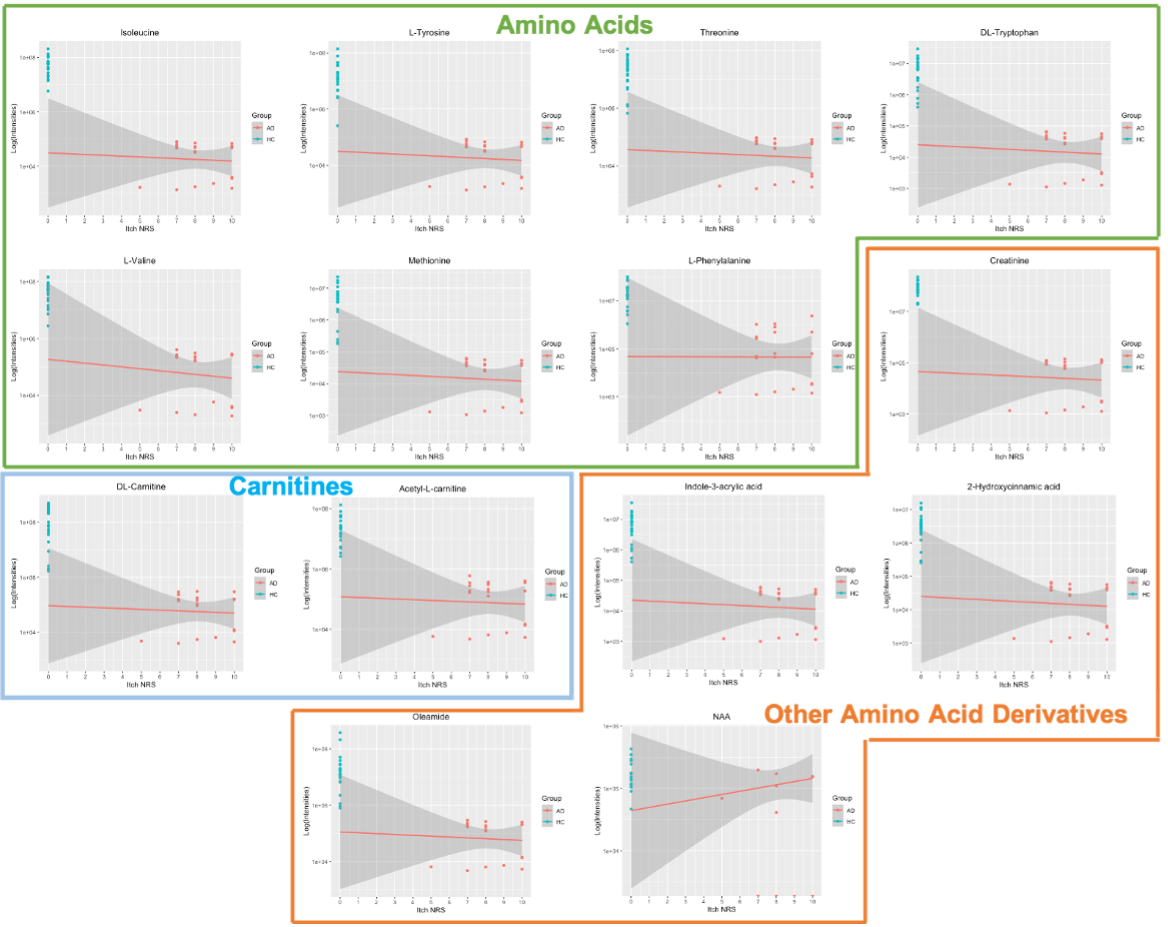

Supplement: Figure S3 — Scatterplots of Log(intensities) vs. itch numerical rating scores (INRS) of selected metabolites. Scatterplots of Log(intensities) vs. INRS scores of significantly decreased metabolites were not found to be significantly correlated with INRS scores. [file Image_3.jpeg]

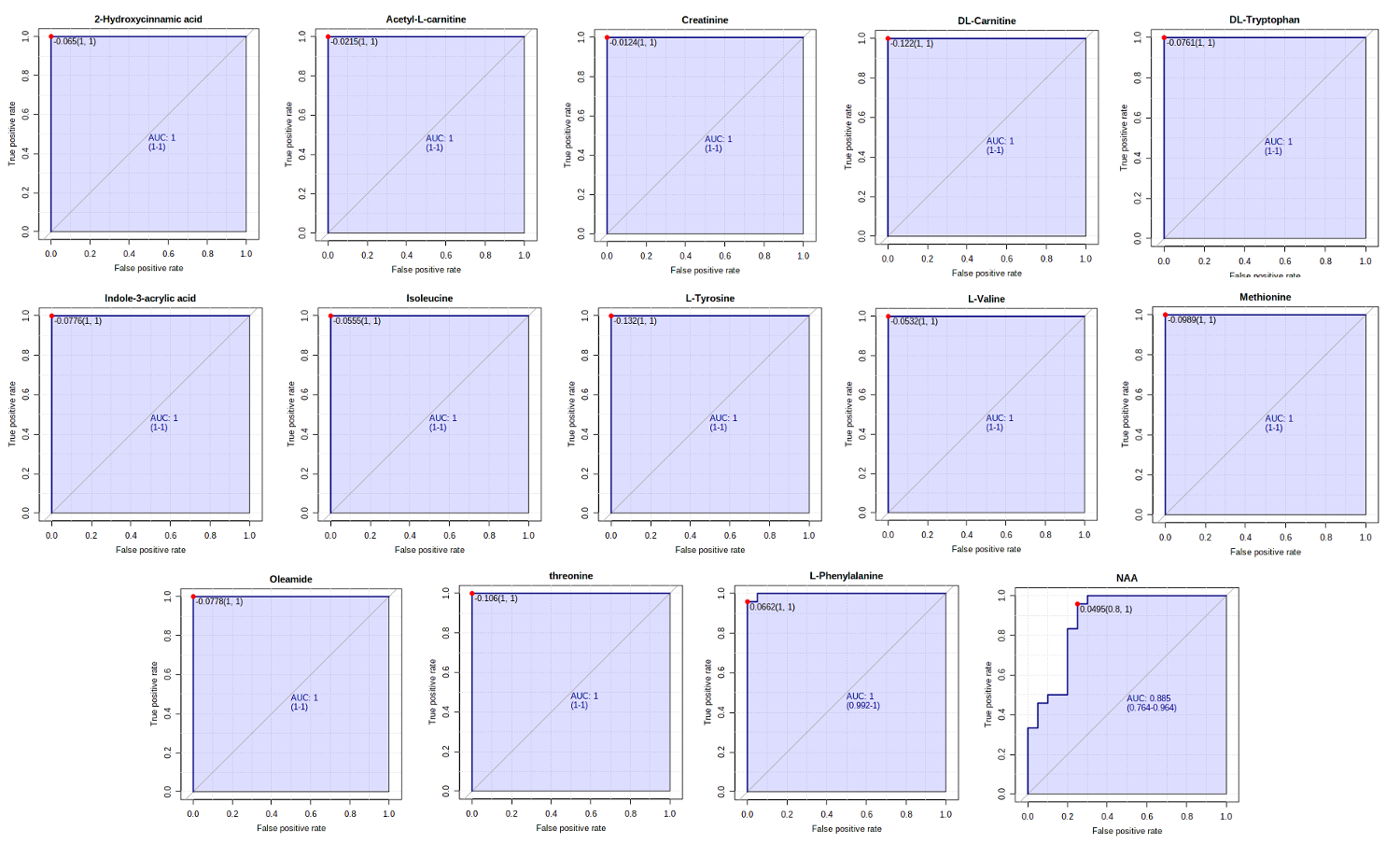

Supplement: Figure S4 — Selected receiver operating characteristic (ROC) curves for biomarker analysis in unmatched patients. Selected ROC curves for metabolites with area under the curve (AUC) values equal to or approximately equal to 1.0, indicating their strong performance potentials as biomarkers. [file Image_4.jpeg]

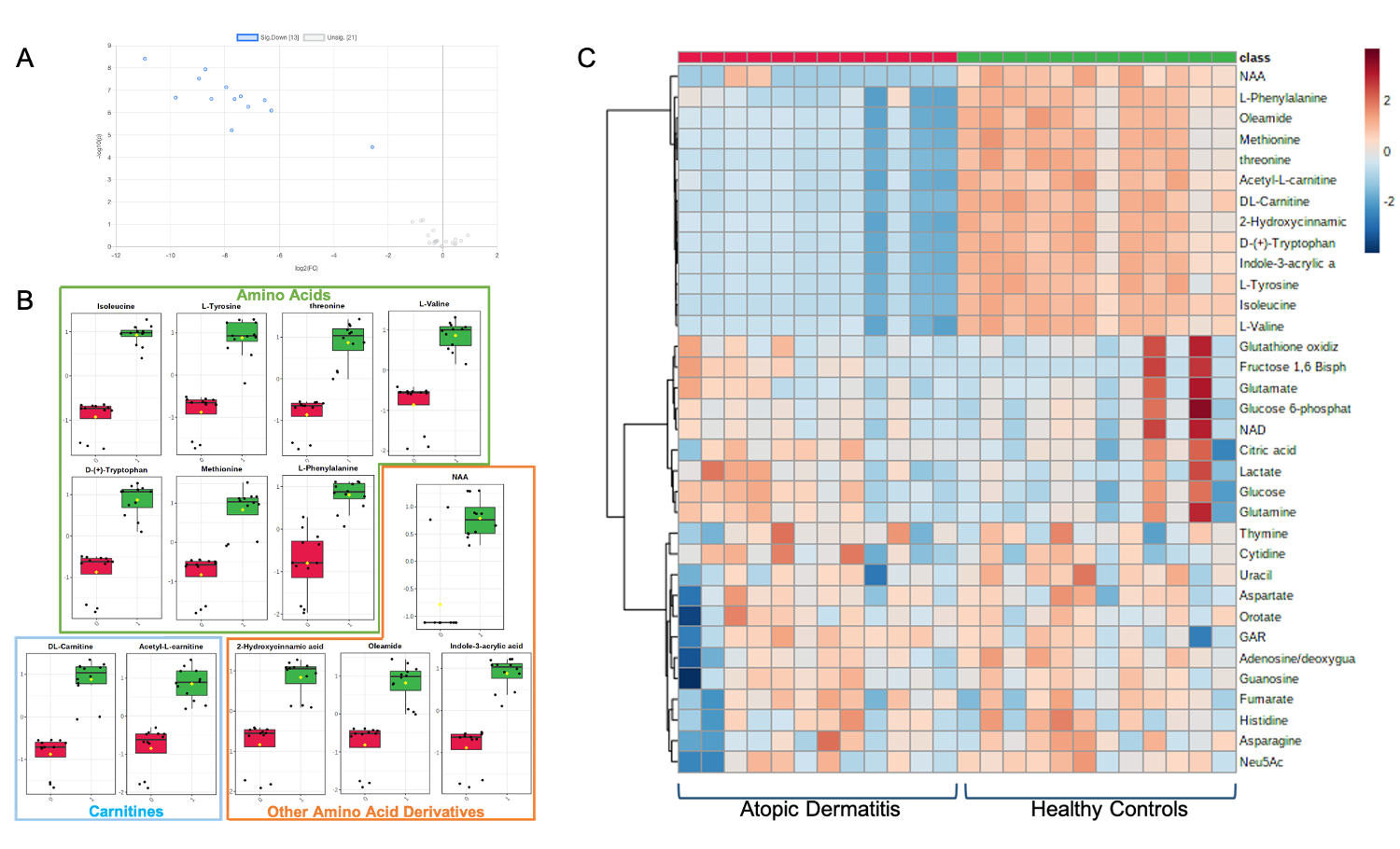

Supplement: Figure S5 — Metabolites with significant alterations in matched AD vs HC patients. (A) Volcano plot of significant fold change distribution of selected metabolites in AD versus HC (blue=significantly downregulated). (B) Boxplot summaries of the peak intensities of the significant metabolites (red=AD, green=HC). (C) Heatmap of the differential metabolites quantified in plasma in AD vs HC individuals using Euclidian distance measure and Ward clustering algorithm. [file Image_5.jpeg]
