# Supplementary material for: Integrated plasma metabolomic and cytokine analysis reveals a distinct immunometabolic signature in atopic dermatitis
Source: Front Immunol. 2024 Mar 15;15:1354128. doi: 10.3389/fimmu.2024.1354128 (PMC10978712; doi:10.3389/fimmu.2024.1354128)
Supplement: Supplementary file 7 [file Table_1.docx]

**Table S1: Demographics of pre-matched and matched atopic dermatitis and healthy control subjects**

| Characteristics | AD patients, metabolomics | HC patients, metabolomics | P-value^2^ | AD patients, cytokine | HC patients, cytokine | P-value^2^ |
| --- | --- | --- | --- | --- | --- | --- |
| Pre-matched |  |  |  |  |  |  |
| Number | 20 | 24 |  | 19 | 33 |  |
| Age in years^1^ | 40.3 ± 17.4 | 47.6 ± 16.6 | .16 | 40.7 ± 17.6 | 45.8 ± 16.3 | 0.30 |
| Gender, n (%) |  |  |  |  |  |  |
| Male | 6 (30.0%) | 6 (25.0%) | .75 | 6 (31.6%) | 10 (30.3%) | 0.92 |
| Female | 14 (70.0%) | 18 (75.0%) |  | 13 (68.4%) | 23 (69.7%) |  |
| Race |  |  |  |  |  |  |
| Caucasian | 5 (25.0%) | 8 (33.3%) | 0.83 | 5 (26.3%) | 12 (36.4%) | 0.69 |
| AA | 13 (65.0%) | 14 (58.3%) |  | 12 (63.2%) | 19 (57.6%) |  |
| Other | 2 (10.0%) | 2 (8.3%) |  | 2 (10.5%) | 2 (6.1%) |  |
| Itch NRS^1^ | 8.2 ± 1.4 | 0 ± 0 | <.0001 | 7.9 ± 1.6 | 0 ± 0 | <.0001 |
| Matched |  |  |  |  |  |  |
| Number | 12 | 12 | 1 |  |  |  |
| Age in years^1^ | 40.2 ± 17.7 | 40.7 ± 17.9 | .95 |  |  |  |
| Gender, n (%) |  |  |  |  |  |  |
| Male | 3 (25.0%) | 3 (25.0%) | 1 |  |  |  |
| Female | 9 (75.0%) | 9 (75.0%) |  |  |  |  |
| Race |  |  |  |  |  |  |
| Caucasian | 3 (25.0%) | 3 (25.0%) | 1 |  |  |  |
| AA | 7 (58.3%) | 7 (58.3%) |  |  |  |  |
| Other | 2 (16.7%) | 2 (16.7%) |  |  |  |  |
| Itch NRS^1^ | 8.1 ± 1.3 | 0 ± 0 | <.0001 |  |  |  |

Abbreviations: AA, African American; AD, atopic dermatitis; HC, healthy control; NRS, Numeric Rating Scale.

^1^Values are presented as the means ± standard deviation. ^2^P-value was calculated by unpaired-Wilcoxon test, χ^2^ test, and Fisher’s exact test.

**Table S2: Physical and chemical characteristics of metabolites.**

See Excel sheet.

**Table S3: List of tested metabolites**

| Isoleucine | 2-Hydroxycinnamic acid | Thymine | Histidine |
| --- | --- | --- | --- |
| L-Tyrosine | Acetyl-L-carnitine | Asparagine | Orotate |
| DL-Carnitine | Oleamide | Glucose | Glutamate |
| L-Valine | N-acetylaspartate | Guanosine | Glutamine |
| Indole-3-acrylic acid | L-Phenylalanine | Glycineamide ribonucleotide | Glutathione oxidized |
| Threonine | Uracil | Glucose 6-phosphate | Aspartate |
| D-(+)-Tryptophan | Adenosine/deoxyguanosine | Citric acid | Cytidine |
| Methionine | Neu5Ac | NAD | Fructose 1,6 Bisphosphate |
| Fumarate | Lactate |  |  |

**Table S4: Biomarker identification and analysis based on receiver operating characteristic (ROC) curves in unmatched patients**

| **Name** | **AUC^1^** | **T-tests** | **log_2_(FC)** |
| --- | --- | --- | --- |
| 2-Hydroxycinnamic acid | 1.0 | 7.80E-16 | -7.0429 |
| Acetyl-L-carnitine | 1.0 | 3.47E-16 | -7.3474 |
| Creatinine | 1.0 | 1.53E-21 | -10.339 |
| DL-Carnitine | 1.0 | 2.60E-17 | -10.501 |
| DL-Tryptophan | 1.0 | 1.46E-17 | -8.0298 |
| Indole-3-acrylic acid | 1.0 | 6.99E-18 | -8.3328 |
| Isoleucine | 1.0 | 5.75E-24 | -10.729 |
| L-Tyrosine | 1.0 | 9.59E-19 | -9.5277 |
| L-Valine | 1.0 | 8.43E-17 | -8.3346 |
| Methionine | 1.0 | 1.09E-15 | -7.7366 |
| Oleamide | 1.0 | 1.90E-14 | -8.1163 |
| threonine | 1.0 | 2.15E-18 | -9.4246 |
| L-Phenylalanine | 0.99792 | 9.97E-13 | -6.2743 |
| NAA | 0.87917 | 9.51E-09 | -1.9275 |
| Adenosine/deoxyguanosine | 0.7875 | 0.006879 | -0.84486 |

^1^Selected area under the curves (AUCs) as measures of performance for different biomarker candidates. Metabolites with AUC values closest or equal to 1.0 have the highest sensitivities and specificities and therefore serve as the best predicted biomarkers.

FC, fold change; NAA, N-acetylaspartic acid.

**Table S5: Metabolites with significant fold changes ≥2 in matched AD vs HC patients**

|  | **Metabolite description** | **Fold change** | **P-value^1^** | **Q-value^2^** |
| --- | --- | --- | --- | --- |
| Isoleucine | amino acid | 0.00050943 | 3.84E-09 | 1.31E-07 |
| L-Tyrosine | amino acid | 0.0023686 | 1.12E-08 | 1.91E-07 |
| threonine | amino acid | 0.0020185 | 2.93E-08 | 3.32E-07 |
| Indole-3-acrylic acid | AA (tryptophan) derivative | 0.0040397 | 7.12E-08 | 6.05E-07 |
| Acetyl-L-carnitine | AA (lysine) derivative | 0.005832 | 1.85E-07 | 1.03E-06 |
| DL-Carnitine | AA (lysine) derivative | 0.0011156 | 2.08E-07 | 1.03E-06 |
| L-Valine | amino acid | 0.0027703 | 2.37E-07 | 1.03E-06 |
| D-(+)-Tryptophan | amino acid | 0.0049931 | 2.43E-07 | 1.03E-06 |
| 2-Hydroxycinnamic acid | AA (phenylalanine/tyrosine) derivative | 0.010712 | 2.72E-07 | 1.03E-06 |
| Methionine | amino acid | 0.0070557 | 5.31E-07 | 1.81E-06 |
| Oleamide | fatty amide | 0.012714 | 7.94E-07 | 2.45E-06 |
| L-Phenylalanine | amino acid | 0.0046374 | 5.99E-06 | 1.70E-05 |
| NAA | AA (aspartate) derivative | 0.16623 | 3.39E-05 | 8.88E-05 |

AA, amino acid; NAA, N-acetylaspartic acid.

^1^P-value based on unpaired t-test; ^2^Q-value, false discovery rate adjusted p-values.

**Table S6: Biomarker identification and analysis based on receiver operating characteristic (ROC) curves in matched patients**

| **Name** | **AUC^1^** | **P-value** | **log_2_(FC)** |
| --- | --- | --- | --- |
| 2-Hydroxycinnamic acid | 1.0 | 9.14E-08 | -6.2668 |
| Acetyl-L-carnitine | 1.0 | 4.37E-08 | -7.2091 |
| D-(+)-Tryptophan | 1.0 | 5.07E-09 | -7.3575 |
| DL-Carnitine | 1.0 | 2.73E-09 | -9.9174 |
| Indole-3-acrylic acid | 1.0 | 2.55E-09 | -7.6404 |
| Isoleucine | 1.0 | 2.13E-12 | -10.516 |
| L-Tyrosine | 1.0 | 2.75E-09 | -8.6793 |
| L-Valine | 1.0 | 1.46E-08 | -7.9558 |
| Methionine | 1.0 | 1.79E-07 | -7.3106 |
| Oleamide | 1.0 | 3.89E-07 | -6.1911 |
| threonine | 1.0 | 7.51E-09 | -9.265 |
| L-Phenylalanine | 0.99306 | 9.88E-07 | -5.6684 |
| NAA | 0.89583 | 2.05E-06 | -1.9427 |
| Uracil | 0.72917 | 0.04105097 | -0.70613 |
| Adenosine/deoxyguanosine | 0.72222 | 0.04328854 | -0.62564 |

^1^Selected area under the curves (AUCs) as measures of performance for different biomarker candidates. Metabolites with AUC values closest or equal to 1.0 have the highest sensitivities and specificities and therefore serve as the best predicted biomarkers.

FC, fold chang; NAA, N-acetylaspartic acid.

**Table S7: Statistically significant metabolic pathways^1^**

|  | Total compounds | Hits | P-value | Holm adjust | Q-value^2^ | Impact |
| --- | --- | --- | --- | --- | --- | --- |
| Catecholamine Biosynthesis | 14 | 1 | 9.59E-19 | 4.41E-17 | 4.41E-17 | 0 |
| Threonine and 2-Oxobutanoate Degradation | 13 | 1 | 2.15E-18 | 9.69E-17 | 4.95E-17 | 0 |
| Oxidation of Branched Chain Fatty Acids | 22 | 2 | 1.58E-17 | 6.97E-16 | 1.50E-16 | 0 |
| Beta Oxidation of Very Long Chain Fatty Acids | 13 | 2 | 1.58E-17 | 6.97E-16 | 1.50E-16 | 0.049458 |
| Tyrosine Metabolism | 55 | 3 | 2.54E-17 | 1.07E-15 | 1.50E-16 | 0 |
| Fatty Acid Metabolism | 40 | 1 | 2.60E-17 | 1.07E-15 | 1.50E-16 | 0 |
| Carnitine Synthesis | 16 | 1 | 2.60E-17 | 1.07E-15 | 1.50E-16 | 0 |
| Mitochondrial Beta-Oxidation of Long Chain Saturated Fatty Acids | 24 | 1 | 2.60E-17 | 1.07E-15 | 1.50E-16 | 0 |
| Valine, Leucine and Isoleucine Degradation | 51 | 1 | 8.43E-17 | 3.20E-15 | 3.88E-16 | 0 |
| Phenylalanine and Tyrosine Metabolism | **25** | **3** | **5.08E-16** | **1.83E-14** | **2.12E-15** | **0.22124** |
| Glycine and Serine Metabolism | 50 | 2 | 3.27E-15 | 1.15E-13 | 1.25E-14 | 0 |
| Aspartate Metabolism | **34** | **5** | **9.72E-08** | **3.31E-06** | **3.44E-07** | **0.8** |
| Selenoamino Acid Metabolism | 28 | 1 | 0.0068787 | 0.227 | 0.019776 | 0 |
| Methionine Metabolism | 39 | 1 | 0.0068787 | 0.227 | 0.019776 | 0 |
| Betaine Metabolism | 18 | 1 | 0.0068787 | 0.227 | 0.019776 | 0 |
| Amino Sugar Metabolism | 31 | 1 | 0.020538 | 0.61615 | 0.055575 | 0.086538 |

^1^Bolded rows represent the highest-impact significant pathways discovered on SMPDB; ^2^Q-value, false discovery rate adjusted P-values.

Holm adjust, Holm-Bonferroni adjusted P-values.
